# Remarks on the Efficacy of Matico as a Styptic and Astringent, with Additional Cases, Mode of Exhibition, &c.

**Published:** 1844-11-02

**Authors:** Thomas Jeffreys


### RECORD OF MEDICAL SCIENCE.

*Remarks on the Efficacy of Matico as a styptic and astringent, with additional cases, mode of exhibition, &c.* By Thomas Jeffreys, M. D.

Matico is the Piper angustifolium of the Flora Peruviana, and possesses, it appears, very great astringent and styptic properties, owing to which it is held in great esteem in the western parts of South America. Having accidentally heard of its virtues some years ago, Dr. Jeffreys procured specimens of the plant from America, and on testing it in cases of hæmorrhage, found that it really was a very valuable agent. The first account which Dr. Jeffreys gave of his researches, appeared in the Lancet of January 5th, 1839. Since then, not only has he had additional opportunities of using the plant, but he has also been able to collect evidence in favor of it from many well-known medical practitioners whom he had summoned to his assistance in his inquiries. Among these gentlemen we may name Dr. W. Monro, of the Dundee Infirmary, and Dr. Watson and Mr. W. H. Bainbridge, of Liverpool. Matico may be used externally or internally, but it is principally in external cases of hæmorrhage that it appears to have been beneficial. Dr. Jeffreys relates several cases of obstinate hæmorrhage from leech-bites—the ablation of nævi, incisions, &c.—in which the flow of blood was arrested, by applying the under side of the leaf to the bleeding surface. The upper side of the leaf does not appear to present the styptic property to the same extent. It has been used internally, with success, in cases of hæmatemesis, hæmorrhage from the intestines, menorrhagia; and as a lotion, in gonorrhœa, vaginitis, &c. From an analysis made by Mr. Clay, of Liverpool, it seems that it contains a considerable quantity of gallic acid, which, no doubt, is the active principle. It may thus be assimilated to the other vegetable astringents which are already known to us; perhaps, more especially to monœsia and paulinia, two powerful vegetable astringents which have lately been introduced into France, also from South America. Whether matico is a more powerful astringent than those of which we are already in possession, it remains for time to prove. Matico is either applied in substance to the bleeding part, or used as an infusion or decoction. The infusion is made by macerating for two hours an ounce of the leaves in a pint of boiling water; the decoction, by boiling for ten or fifteen minutes an ounce or more of the leaves in the same quantity of water. The dose of either is two tablespoonfuls twice or thrice a day. There are two kinds of matico forwarded to this country, one green, the other yellow. They appear to be the same plant, but at different periods of its growth. The yellow is the most efficacious. There is a spurious herb in the market which is quite inefficacious, so that it is necessary to be very careful in procuring it.—*London Lancet.*

